# Perceptions about the cause of schizophrenia and the subsequent help seeking behavior in a Pakistani population – results of a cross-sectional survey

**DOI:** 10.1186/1471-244X-8-56

**Published:** 2008-07-17

**Authors:** Syed Nabeel Zafar, Reema Syed, Sarah Tehseen, Saqib A Gowani, Sana Waqar, Amina Zubair, Wajeeha Yousaf, Akbar J Zubairi, Haider Naqvi

**Affiliations:** 1 Aga Khan University, Stadium Road, Karachi 74800, Pakistan; 2 Assistant Professor, Department of Psychiatry, Aga Khan University, Stadium Road, Karachi 74800, Pakistan

## Abstract

**Background:**

There is a cultural variability around the perception of what causes the syndrome of schizophrenia. Generally patients with schizophrenia are considered dangerous. They are isolated and treatment is delayed. Studies have shown favorable prognosis with good family and social support, early diagnosis and management. Duration of untreated psychosis is a bad prognostic indicator. We aimed to determine the perceptions regarding the etiology of schizophrenia and the subsequent help seeking behavior.

**Methods:**

This cross-sectional study was carried out on a sample of 404 people at the out patient departments of Aga Khan University Hospital Karachi. Data was collected via a self-administered questionnaire. Questions were related to a vignette of a young man displaying schizophrenic behavior. Data was analyzed on SPSS v 14.

**Results:**

The mean age of the participants was 31.4 years (range = 18–72) and 77% of them were males. The majorities were graduates (61.9%) and employed (50%). Only 30% of the participants attributed 'mental illness' as the main cause of psychotic symptoms while a large number thought of 'God's will' (32.3%), 'superstitious ideas' (33.1%), 'loneliness' (24.8%) and 'unemployment' (19.3%) as the main cause. Mental illness as the single most important cause was reported by only 22%. As far as management is concerned, only 40% reported psychiatric consultation to be the single most important management step. Other responses included spiritual healing (19.5%) and Sociachanges (10.6) while 14.8% of respondents said that they would do nothing. Gender, age, family system and education level were significantly associated with the beliefs about the cause of schizophrenia (p < 0.05). While these variables plus 'religious inclination' and 'beliefs about cause' were significantly associated with the help seeking behavior of the participants.

**Conclusion:**

Despite majority of the study population being well educated, only a few recognized schizophrenia as a mental illness and many held superstitious beliefs. A vast majority of Pakistanis have non-biomedical beliefs about the cause of schizophrenia. Their help seeking behavior in this regard is inappropriate and detrimental to the health of schizophrenic patients. Areas for future research have been identified.

## Background

Schizophrenia is a severe mental illness characterized by fundamental disturbances in thoughts, emotions and perceptions. Even though not frequent it is amongst the most burdensome and costly diseases of the world[1]. WHO reports it as the 8th leading cause of DALYS (disability adjusted life years) in the age group of 15 to 44 years [1]. Schizophrenic patients place a significant burden on mental health delivery system through chronic disability, social dysfunction, frequent hospitalizations, substance abuse and overall poor compliance[2]. Furthermore the families and caregivers of schizophrenic patients also bear the weight of its negative social and financial influences. Schizophrenia imposes huge economic impact on individuals, families, and communities [3]. Hence it is a disease that affects not only the individual but bears an impact on the whole society.

Functional outcome in patients with schizophrenia is influenced by many factors including duration of illness, symptoms, sex, early detection and pre-morbid functioning [4]. Attitudes of family members towards schizophrenics is an independent predictor of social adjustment in these individuals [5]. Cultural features, socioeconomic level of the region and education level are also important among the factors influencing the incidence, course and duration of untreated psychosis of this disease. The duration of untreated psychosis or DUP has recently emerged as an important prognostic factor for first episode psychosis [6,7].

Unfortunately, lay public across different cultures hold highly variable beliefs regarding the cause of schizophrenia. In developed countries social factors are considered to be the most important causative agents for schizophrenia [8]. In developing countries like India and Morocco a vast majority of people attributed the schizophrenic symptoms to supernatural phenomena, drug use, stressful life events, heredity or personality deficiencies [8,9]. These beliefs about the causation of schizophrenia are the primary forces that lead the public towards variable treatment modalities and delays in professional medical treatment and hence alter the outcome of this illness [10].

Pakistan is a developing country where mental illnesses are widely perceived to have supernatural causes[11] and are stigmatized even by the educated masses [12].

Traditional healers along with psychiatric services are the main mental health service providers and the number of trained mental health professionals is small as compared to the population demands [11]. Thus, seeking the help of faith healers is often a first step in the management of schizophrenia. This leads to significant under detection and long delays in medical help for those in need. A recent study conducted in Pakistan showed that lack of awareness and false beliefs accounted for 61% of the cases of delayed consultation for first episode psychosis [13].

There is a serious dearth of data in this regard from Pakistan. Very few studies have been conducted to assess the beliefs of the general public regarding the cause of schizophrenia [13] and the help seeking behavior of these people towards a person displaying schizophrenic or psychotic behavior has never been explored. We therefore conducted a cross-sectional survey exploring the beliefs regarding cause of Schizophrenia and subsequent help seeking behavior in a Pakistani population.

## Methods

### Sample and settings

We designed a questionnaire based cross sectional study, which was conducted at the Aga Khan University Hospital (AKUH) Karachi, Pakistan. AKUH is one of the largest private tertiary hospitals in the country, providing high quality health services to people from all over Karachi and Pakistan. This university hospital has 542 beds in operation and provides services to over 38,000 hospitalized patients and to over 500,000 outpatients annually.

The study was conducted in the waiting areas of the Community Health Centre (CHC), consulting clinics (CCs) and inpatient family waiting areas of the hospital. The CCs constitute the main out patient department of the hospital and offers specialist services mainly to people belonging to the middle and high socioeconomic classes, The CHC however, is a primary health care center and provides family medicine and specialist services at a much subsidized rate and is therefore attended by people belonging to all socioeconomic classes. The waiting areas of psychiatry clinics and wards are located elsewhere and data was not collected from these locations. AKUH is visited by people from all over Pakistan. Most attendants at AKUH belong to Muslim middle class, educated families. However this population is culturally diverse owing to the presence of different ethnic communities in the city.

We approached all individuals above 18 years of age for participation in the study. The Study was conducted from 18^th ^to 22^nd ^December 2006. Written informed consent was taken from all the participants. In order to maintain confidentiality, no identifiable personal information was recorded. A group of fourth year medical students interviewed the subjects. Medical staff, medical students, attendants of psychiatric patients, people who could not read Urdu (the national language of Pakistan) or who had difficulty in understanding the questionnaire due to cognitive impairment and subjects under the age of 18 years were excluded from the study. In order to maintain the standardization, a training session for the interviewers was organized prior to data collection.

With a response rate of 87% we enrolled 404 participants. The commonest reason for refusal was lack of time and in the case of females, the male partner not being present to give consent, which is a cultural norm in some of our communities.

### The Questionnaire

We used a self administered questionnaire designed by the investigators after an extensive literature search [8,14,15] (Additional file 1). Prior to the study it was pre-tested on a sample of twenty individuals. No major changes were required and this data was discarded. The whole of the questionnaire was developed in Urdu, the national language of Pakistan.

It was divided into three parts. The first dealt with the demographic profile of the subjects (age, sex, religious inclination, marital status, education and profession). The second comprised of a case scenario of a 24 yr old single, unemployed young man who was showing behavioral changes and psychotic symptoms characteristic of paranoid schizophrenia such as persecutory delusions and auditory hallucinations. The participants were asked what, in their view, was the reason for this man's condition. A list of 22 choices (randomly arranged) was given below the scenario. This list was derived from previous studies showing the general public's belief about the cause of psychotic symptoms[8,15] A four point likert scale (main reason, possible reason, not likely a reason and definitely not a reason) allowed the participants to rate each possibility according to its subjective importance. They were then asked to select the single most important cause of these symptoms.

The third section asked the subjects to state their reaction if one of their family members or anyone close to them showed similar symptoms. They were given a list of 15 interventions (randomly arranged) to consider when faced with such a situation. A three point likert scale (will definitely do, will consider doing, will not do at all) was provided for each option. At the end they were asked to choose the single most important step that they would take.

### Ethical considerations

Ethical approval was granted by the department of Psychiatry, faculty office building, Aga Khan University Hospital, Karachi. The study was in accordance to the Helsinki declaration. Each participant was verbally explained the nature of the research, its benefits and disadvantages. Confidentiality was assured and maintained and no information that could link an individual to the data was recorded. Written informed consent was taken from each person before the administration of the questionnaire and they were told that they could withdraw from the study at any time without giving a reason.

### Data management and statistical analysis

Data was double entered on Epi-Info version 6 and then exported to SPSS version 14 for data management and analysis. Descriptive analyses were performed. Means and standard deviations were calculated for continuous variables while proportions were calculated for discrete ones and the results were tabulated. The responses were categorized into six categories for the cause of schizophrenia (biological causes, psychosocial stressors, social issues, personality issues, religious reasons and superstitious beliefs) and five categories for 'help seeking behavior' (professional medical help, religious remedies, social interventions, magic/exorcism related, no help seeking behavior). Univariate logistic regression analyses were then performed on each of these categories against the demographic variables to identify associations and calculate odds ratios. The five categories of the help seeking behavior were also separately analyzed against the six categories of the cause of schizophrenia. The Odds ratio (OR) and confidence interval (CI) for each variable were calculated.

## Results

The demographic profile of the participants is demonstrated in table 1. The mean age of the participants was 31.35 (± 9.93) with a gender distribution of 77% males and 23% females. Most of the participants were well educated and 62% of them had at least a bachelor's degree. A large majority of participants were Muslim by religion (92%).

**Table 1:** Demographic details of participants (n = 404)

**Variables**		**N (%)**
Sex	male	311 (77.0)
	female	93 (23.0)
Education	none	10 (2.50)
	Primary (grades 1 to 5)	22 (5.40)
	Secondary (grades 6 to 10)	36 (8.90)
	College (grades 11,12)	86 (21.3)
	Graduate (bachelors degree)	250 (61.9)
Ethnicity	Urdu speaking	195 (48.3)
	Sindhi	40 (9.90)
	Punjabi	47 (11.6)
	Pathan	37(9.20)
	Baluchi	7 (1.70)
Employment status	Unemployed	17 (4.20)
	Employed	202 (50.0)
	Retired	5 (1.2)
	Housewife	124 (50)
	Student	64 (15.8)
	Others	66 (16.3)
Marital status	Single	149 (36.9)
	Married	239 (59.2)
	Divorced/separated	10 (2.50)
	Widow/widower	6 (1.50)
Religion	Islam	373 (92.3)
	Christianity	12 (3.00)
	Hinduism	17 (4.2)
	Zoroastrian	2 (0.50)
Religious orientation	Not religious	14 (3.5)
	Mildly religious	70 (17.3)
	Moderately religious	223 (55.2)
	Very religious	97 (24.0)
Family System	Joint	306 (75.7)
	Nuclear	98 (24.3)

Age Range = 18 – 72 Mean = 31.35 (± 9.93)

### Cause of schizophrenia

The participants gave highly variable responses to the question that assessed their opinion about the cause of schizophrenia demonstrating that many held multiple explanatory models to this. These responses are shown in table 2 and have been divided for tabulation and analysis purposes into six categories namely biological causes, psychosocial causes, social issues, personality issues, religious reasons and superstitious beliefs.

**Table 2:** Participant's beliefs concerning the cause of schizophrenic disorders (divided into six categories) (n = 404)

	**Main reason**	**Possible reason**	**Not likely**	**Definitely not**
	**N (%)**	**N (%)**	**N (%)**	**N (%)**
**Biological causes**				
Mental illness	121 (30.0)	122 (30.2)	67 (16.6)	94 (23.3)
Hereditary/genetic	36 (8.9)	122 (30.3)	93 (23.1)	152 (37.7)
Weak mental constitution	91 (22.5)	143 (35.4)	64 (15.8)	106 (26.2)
**Psychosocial stressors**				
Marital problems	44 (11.0)	154 (38.4)	60 (15.0)	143 (35.7)
Work stress	70 (17.3)	149 (36.9)	83 (20.5)	102 (25.2)
Busy lifestyle	35 (8.7)	102 (25.2)	113 (28.0)	154 (38.1)
Unemployment	78 (19.3)	122 (30.2)	67 (16.6)	137 (33.9)
Failure in love	62 (15.4)	143 (35.6)	67 (16.7)	130 (32.3)
**Social Issues**				
Loneliness	100 (24.8)	141 (34.9)	62 (15.3)	101 (25.0)
Bad upbringing	38 (9.4)	102 (25.2)	97 (24.0)	167 (41.3)
Sexual abuse during childhood	24 (6.0)	100 (24.9)	97 (24.2)	180 (44.9)
**Personality Issues**				
Anxious personality	69 (17.1)	146 (36.2)	82 (20.3)	106 (26.3)
Attention seeking behavior	44 (10.9)	145 (36.0)	103 (25.6)	111 (27.5)
Alcohol or other addictions	31 (7.7)	117 (29.0)	74 (18.3)	182 (45.0)
**Religious reasons**				
Punishment for sins	63 (15.6)	104 (25.7)	83 (20.5)	154 (38.1)
Fate	36 (8.9)	64 (15.9)	93 (23.1)	210 (52.1)
Gods will	130 (32.3)	67 (16.7)	65 (16.2)	140 (34.8)
**Superstitious beliefs**				
Black magic	37 (9.2)	79 (19.6)	76 (18.9)	211 (52.4)
Taweez	27 (6.7)	79 (19.6)	90 (22.3)	208 (51.5)
Nazar	22 (5.5)	78 (19.4)	101 (25.1)	202 (50.1)
Possessed by bad spirits	27 (6.7)	105 (26.1)	80 (19.9)	190 (47.3)
Extraterrestrial influence	20 (5.0)	57 (14.1)	87 (21.6)	239 (59.3)

Each row totals to 404 (100%)

When asked to give a single most important reason for the condition of the young man in the given scenario only 90 (22.3%) people said that it was due to a mental illness. Other common responses were weak mental constitution (13.6%) and God's will (10.1%). The responses of the remaining 54% of participants were distributed throughout the other choices listed in table 2. When all the responses were categorized into the fore mentioned six categories it was seen that 38.4% people gave a biological cause, 15.6% gave a religious cause, 13.4% stated that it was due to a personality issue, while 12.1% gave a psychosocial stressor as a cause and 8.4% stated other social issues as the cause of the schizophrenic symptoms.

After applying univariate logistic regression analysis it was found that: Females (OR = 1.92, CI = 1.202–3.069), participants living in a nuclear family (OR = 1.79, CI = 1.127–2.830) and those with an age greater than 47 (OR = 2.092, CI = 1.045–4.188) were more likely to give a biological cause. While those who had received only primary level education were 3.3 times (CI = 1.363–8.154) more likely than graduates to give a psychosocial stressor as a reason for the man's condition.

### Help seeking behavior

Table 3 shows the responses of the participants when asked what they would do if someone dear to them was exhibiting such behavior. For tabulation and analysis purposes these responses were divided into five categories: professional medical help, religious remedies, social interventions, magic/exorcism related and no help seeking behavior.

**Table 3:** Help seeking behavior of participants towards a relative with schizophrenia (divided into five categories) (n = 404)

	**will definitely do** **N (%)**	**will consider doing** **N (%)**	**Will definitely not do** **N (%)**
**Professional medical help**			
Take him to a family physician	189 (48.3)	97 (24.8)	105 (26.9)
Take him to a psychiatrist	254 (62.9)	70 (17.3)	80 (19.8)
Take him to a mental hospital	29 (7.2)	47 (11.6)	327 (80.9)
**Religious remedies**			
Take him to a Mazaar*	53 (13.2)	62 (15.4)	286 (71.0)
Take him to an Alim/Imam**	129 (31.9)	107 (26.5)	168 (41.6)
Pray	201 (49.9)	96 (23.8)	106 (26.3)
Sadqa khairaat^α^	223 (55.3)	60 (14.9)	120 (29.8)
**Social Interventions**			
get him married	102 (25.2)	112 (27.7)	190 (47.0)
get him employed	161 (39.9)	113 (28.0)	130 (32.2)
change his job	68 (16.8)	162 (40.1)	174 (43.1)
**Magic/Exorcism related**			
exorcize him	63 (15.6)	87 (21.5)	253 (62.6)
go to an Amil^∞^	33 (8.2)	72 (17.8)	297 (73.5)
taweez^ϒ^	42 (10.4)	69 (17.1)	292 (72.3)
**No help seeking behavior**			
talk to him	223 (55.2)	86 (21.3)	95 (23.5)
nothing, rest	173 (42.9)	118 (29.3)	112 (27.8)

* tomb of a religious person, **A religious leader, ^α ^sacrifice in the name of God, ^∞ ^A person who does black magic, ^ϒ ^An item worn around the neck or arm in order to protect against evil.

Each row totals to 404 (100%)

In response to the question asking the participants to give the single most immediate and important management option for the young man's condition 164 (40.6%) of them said that they would take him to a psychiatrist. This response was, by far, the most popular response followed by 'will take him to a family physician' (9.7%), 'do nothing and talk to him' (8.9%), 'offering sadqa/khairaat' i.e. sacrifice in the name of God, (7.4%) and 'pray for him' (6.7%). Categorically the most common response was in the seeking professional medical help category (52.2%) followed by religious remedies (19.3%), no help seeking behavior (14.9%), social alterations (10.6%) and magic exorcism related management steps (3.0%).

Upon univariate analysis it was seen that those who were more likely to seek professional medical help were those who were living in a nuclear family (OR = 2.29, CI = 1.420–3.694), were moderately religious (OR = 1.72, CI = 1.066–2.787), females (OR = 2.17, CI = 1.339–3.532) and those who gave the cause of schizophrenia to be biological (OR = 12.93, CI = 6.377–26.255) or being a personality issue (OR = 3.23, CI = 1.462–7.223).

People more likely to seek a religious remedy were very religious (OR = 2.32 CI = 1.28–4.237), less educated (illiterate OR = 3.84, CI = 1.033–14.258; primary OR = 2.69, CI = 1.026–7.035; higher secondary OR = 1.86, CI = 1.017–3.40), living in a joint family system (OR = 2.281, CI = 1.121–4.642) and those who had given a religious cause for schizophrenia (OR = 6.56, CI = 3.156–13.630).

Those who exhibited no help seeking behavior were more likely to be young (age group 17–26, OR = 8.49, CI = 1.119–64.332; age group 27–36, OR = 9.134, CI = 1.206–69.192) males (OR = 2.581, CI = 1.132–5.887) and those who had given any non-biological reason as a cause of schizophrenia (p = 0.010).

## Discussion

It is well known that the general population of most developing countries attributes a wide range of non-biomedical beliefs to the cause of schizophrenia and other psychotic disorders [16,17]. In fact people in developed countries have also displayed such beliefs [15,18]. Therefore the wide variety and confused nature of beliefs found in our sample comes as no surprise. No substantial study has been conducted in Pakistan in this regard to compare the data of our study with. However, studies from India, Pakistan's neighboring country and similar in terms of cultural, social and traditional values and beliefs have shown that even urban and adequately educated families of India hold supernatural beliefs about the cause and management of schizophrenia [16] and majority of the psychiatric patients undergo traditional treatment [19].

The duration of untreated psychosis or DUP is the time from manifestation of the first psychotic symptom to initiation of adequate treatment. It has been shown to have a significant association with the outcome of the disease and is emerging as an important factor in the management of schizophrenic patients [6,7]. Recent evidence also suggests a possibility of an association between DUP and homicide [20] making it an even more important variable to look at.

The different causes attributed to schizophrenia by our study sample are shown in table 2.

This is important, as it is this belief about the cause that drives the direction of treatment. If a person believes that the cause is a supernatural or religious one then one tends to seek faith healing as a remedy for the illness. And thus seeking such help then becomes the first step in the management of most mental disorders [21]. This causes substantial delays in seeking professional treatment, and subsequently leads to a poorer prognosis, higher burden on society and a higher cost of the disease. Our study has demonstrated that people who gave a biological cause for the disease were almost 13 times more likely to seek professional medical help for a relative with schizophrenia than those who gave other reasons for the disease. This highlights how the belief about the cause of schizophrenia can drive the help seeking behavior and thus decrease the duration of untreated psychosis. However, the tables show that people generally have multiple explanatory models to the cause and management of schizophrenia. Previous studies have also demonstrated that relatives of schizophrenic patients often hold multiple, diverse and sometimes contradictory explanatory models of illness [22]. This elicits the complexity of the issue and highlights the need for further local research in this area.

Another problem of holding non-biomedical beliefs is that they also lead to the stigmatization of schizophrenic patients. Schizophrenia is considered to be most stigmatizing of all psychiatric illnesses[23] and these non-biomedical beliefs could be one of the main reasons. This stigmatization adversely affects the self esteem of schizophrenics [24] and acts as a major barrier to recovery[25]. Awareness and Education can reverse this as countless studies have demonstrated the value of good family support in the recovery of schizophrenia[5] and both eastern and western data show how the beliefs of relatives of patients with schizophrenia are different from the general population and this is mainly attributed to their increased awareness about the disease [8,26]. The relationship of non-medical beliefs with stigmatization was not amongst the objectives of this paper and therefore was not studied. However, this relationship is important to understand and should be an avenue for future research.

It is vitally important that these beliefs be changed. Previous studies have shown the importance of education and awareness in changing the beliefs of the general population to the cause of schizophrenia and thus their help seeking behavior[27]. Our study has also demonstrated that less educated people are more likely to have an incorrect perception about schizophrenia. A point to note here that education alone is not sufficient, awareness about the disease is more if not equally important in determining the causal beliefs among the population [28]. Living in a nuclear family system, being of female gender and having a higher age were also significantly associated with stating the correct cause. Most people of Pakistan live in a joint family system in which people tend to adhere to ancient and traditional concepts of diseases and false supernatural explanations are passed down from generation to generation. Awareness programs need to prioritize on the illiterate population, on young people, males and those living in joint family systems to be more effective.

However, the situation is more complex than this and simple awareness or educational interventions have shown to have limited success. A randomized control trial conducted in India showed that a structured educational intervention did significantly decrease the non-biomedical causal explanatory models but failed to decrease the treatment explanatory models [29]. While a review of literature demonstrated that enforcement of biogenetic causal theories amongst the public were linked to negative perceptions of dangerousness and unpredictability which led to fear and increased distancing [30]. Much research on the cultural perceptions of schizophrenia is needed and local data must be sought. The use of universal standardized interventions may not prove to be successful. Local beliefs need to be elicited, discussed and addressed in any intervention aimed at altering causal and treatment explanatory models.

The help seeking behavior of our population as demonstrated by this study is very distressing. Not many people said that they would seek professional medical help for a person displaying schizophrenic or psychotic behavior. This is indeed a grave problem as besides increasing the duration of untreated psychosis, it results in the underreporting of the disease. The correct prevalence of schizophrenia in Pakistan can not be known and thus the seriousness of the condition will always be masked. This prevents large scale and national level strategies from being devised to combat this burdensome and debilitating illness.

An important point to note here is that the sample in our study consists of a large proportion of educated people. This proportion is very different for Pakistan in general. More than 70% of the Pakistani population is illiterate and majority come from the low socioeconomic class. Thus the 40% professional help seeking behavior in our study is a large overstatement. It is unfortunate to note that if amongst a sample of educated people only 30% gave a medical cause and only 40% gave the medical treatment option then the proportions amongst the enormous illiterate population of 120 million maybe extremely unfavorable. Furthermore this study was conducted in a hospital setting and due to social desirability biases, people were generally expected to give a 'seeking medical help' answer. The study population is not representative of the population of Pakistan.

There is a need for further research in this topic and similar studies need to be conducted on more representative samples in order to estimate the true burden of the problem. This is the most important limitation of our study, that it can not be generalized to the whole population. The sample has been taken from a convenient site and thus lacks representativeness. However, the sample serves the purpose of this study which was to give an adequate idea of the beliefs and perceptions of the general population regarding the cause and management of schizophrenia. The purpose of this study was not to estimate exactly how many or what proportion of people have non-biomedical beliefs but to merely demonstrate that a majority of people have incorrect perceptions about schizophrenia and what these perceptions and beliefs are. It was essential to report these finding in order for this issue to be taken seriously and to open doors for future research and intervention

Other limitations of this study include the central tendency bias that exists with all likert scaled questions. No multivariate analyses were performed and thus potential confounding factors were not eliminated. However, this would have been out of the scope of this paper but should be used in future studies in which the objective would be to accurately identify those people with these non-biomedical beliefs and perceptions.

## Conclusion

A vast majority of Pakistanis have many non-biomedical beliefs to the cause of schizophrenia. Their help seeking behavior in this regard is inappropriate and actually detrimental to the health of schizophrenic patients and thus has harmful effects on the society. To decrease the duration of untreated psychosis and limit the burden that schizophrenia has on society, further research is warranted. Effective interventions that that address the non-biomedical causal and treatment explanatory models need to be developed and tested.

## Competing interests

The authors declare that they have no competing interests.

## Authors' contributions

SNZ conceived of the idea of the study, designed it, coordinated it, made the questionnaire, collected data, performed the statistical analysis, conducted literature search and wrote the manuscript. ST and RS conceived the idea of the study, designed it, made the questionnaire, collected data, conducted literature search and helped in writing and editing the manuscript. SAG, SW, AZ, WY and AJZ helped in conceiving the idea of the study and its design and collected data. HN conceived the idea of the study, helped design the questionnaire and reviewed the final manuscript. All authors have approved of the final version to be published.

## Pre-publication history

The pre-publication history for this paper can be accessed here:



## Supplementary Material

Additional file 1 Questionnaire. The questionnaire used in the study was in the Urdu language which is the national language of Pakistan. The English translation of this questionnaire has been provided. Click here for file
