# Supplementary material for: Perceptions about the cause of schizophrenia and the subsequent help seeking behavior in a Pakistani population – results of a cross-sectional survey
Source: BMC Psychiatry. 2008 Jul 17;8:56. doi: 10.1186/1471-244X-8-56 (PMC2483701; doi:10.1186/1471-244X-8-56)
Supplement: Additional file 1 — Questionnaire. The questionnaire used in the study was in the Urdu language which is the national language of Pakistan. The English translation of this questionnaire has been provided. [file 1471-244X-8-56-S1.doc]

TRANSLATED QUESTIONNAIRE – beliefs about the cause of schizophrenia and the subsequent help seeking behavior

| NO | Variable | Response with codes | Code |
| --- | --- | --- | --- |
| DEMOGRAPHY | | | |
| 1 | Age |  |  |
| 2 | Sex | 1. Male  2. Female |  |
| 3 | Religion | 1. Muslim 2. Christian 3. Hindu 4. Zoroastrian 5. others |  |
| 4 | Religious inclination | 1. Not at all religious  2. Somewhat religious  3. Moderately religious  4. Very religious |  |
| 5 | Marital Status | 1. Married  2. Unmarried  3. Widower / widowed  4. Divorced / Separated  5. Others |  |
| 6 | Education | 1. None 2. Can read 3. Primary 4. Secondary 5. Matric 6. High School/Intermediate 7. Graduate/Post-grad |  |
| 7 | Family system | 1. Nuclear  2. Joint  3. Extended |  |
| 8 | Employment status | 1. Unemployed 2. Employed 3. Retired 4. Housewife 5. Student 6. others |  |
| 9 | Ethnicity | 1. Sindhi  2. Punjabi  3. Pathan  4. Baluchi  5. Urdu speaking  6. Memon  7. Others |  |

------CASE SENARIO------ (see end of questionnaire)

What in your opinion is the cause of this behavior?

Please check one box in front of each option

|  |  | Main Reason | Possible reason | Not likely | Definitely not |
| --- | --- | --- | --- | --- | --- |
| 10 | Low IQ |  |  |  |  |
| 11 | Attention seeking behaviour |  |  |  |  |
| 12 | Mental illness |  |  |  |  |
| 13 | Hereditary |  |  |  |  |
| 14 | Marital Issues |  |  |  |  |
| 15 | Work Tension |  |  |  |  |
| 16 | Busy lifestyle |  |  |  |  |
| 17 | Loneliness |  |  |  |  |
| 18 | Anxious personality |  |  |  |  |
| 19 | Fate |  |  |  |  |
| 20 | Alcohol/ Addictions |  |  |  |  |
| 21 | Unemployment |  |  |  |  |
| 22 | Bad upbringing |  |  |  |  |
| 23 | Sexual abuse during childhood |  |  |  |  |
| 24 | Alien influence |  |  |  |  |
| 25 | Black Magic |  |  |  |  |
| 26 | Taweez |  |  |  |  |
| 27 | Nazzar |  |  |  |  |
| 28 | Failure in love |  |  |  |  |
| 29 | Allah’s wish |  |  |  |  |
| 30 | Punishment for sins |  |  |  |  |
| 31 | Possessed by Jin, Evil spirits etc |  |  |  |  |

32. What in your opinion is the single most important cause of this mans condition ______________

What will you do in such a situation?

Please place a check one box in front of each option.

|  |  | Will definitely  do | Will consider it | Definitely not |
| --- | --- | --- | --- | --- |
| 33 | Nothing, tell him to rest |  |  |  |
| 34 | Go to a family physician |  |  |  |
| 35 | Go to a psychiatrist |  |  |  |
| 36 | Counsel him yourself |  |  |  |
| 37 | Go to a Mazar |  |  |  |
| 38 | Pray |  |  |  |
| 39 | Go to an Alim/Imam |  |  |  |
| 40 | Go to an Aamil |  |  |  |
| 41 | Try to dispossess him from evil spirits |  |  |  |
| 42 | Get him married |  |  |  |
| 43 | Get him employed |  |  |  |
| 44 | Change his job |  |  |  |
| 45 | Taweez |  |  |  |
| 46 | Charity (Sadqa khairat) |  |  |  |
| 47 | Take him to a Mental Hospital |  |  |  |

48. Which single option amongst these in your view is the most immediate and important management step ________

**Case scenario**

Following are the translated salient features of the Case scenario (In the formatted questionnaire the case was placed as indicated above)

Mansoor, an unmarried male of age 24 years has been acting strangely for the past few months.

He prefers to stay in isolation and has cut himself off from his close friends.

He has become quite irritable.

He has even wrecked/broken/damaged (created chaos) at his office and at home a few times.

When asked why he has done so he replied by saying that a Godlike voice has instructed him to destroy the Devil.

He says he is being chased by intelligence agencies and thinks that the news on the Television and Radio are related to him.

🡪 Imagine that all of this was being displayed by a relative/close friend of yours and answer the following questions.
